# Pancreatitis, very early compared with normal start of enteral feeding (PYTHON trial): design and rationale of a randomised controlled multicenter trial

**DOI:** 10.1186/1745-6215-12-73

**Published:** 2011-03-10

**Authors:** Olaf J Bakker, Hjalmar C van Santvoort, Sandra van Brunschot, Usama Ahmed Ali, Marc G Besselink, Marja A Boermeester, Thomas L Bollen, Koop Bosscha, Menno A Brink, Cornelis H Dejong, Erwin J van Geenen, Harry van Goor, Joos Heisterkamp, Alexander P Houdijk, Jeroen M Jansen, Thom M Karsten, Eric R Manusama, Vincent B Nieuwenhuijs, Bert van Ramshorst, Alexander F Schaapherder, George P van der Schelling, Marcel BM Spanier, Adriaan Tan, Juda Vecht, Bas L Weusten, Ben J Witteman, Louis M Akkermans, Hein G Gooszen

**Affiliations:** 1 Dept. of Surgery, University Medical Center Utrecht, HP G04.228, PO 85500, 3508 GA Utrecht; The Netherlands; 2 Dept. of Surgery, Radboud University Nijmegen Medical Centre, HP 630, PO 9101, 6500 HB Nijmegen; The Netherlands; 3 Dept. of Surgery, Academic Medical Center Amsterdam, PO 22660, 1100 DD Amsterdam; The Netherlands; 4 Dept. of Radiology, St Antonius Hospital Nieuwegein, PO 2500, 3430 EM Nieuwegein; The Netherlands; 5 Dept. of Surgery, Jeroen Bosch Hospital, PO 90153, 5200 ME Den Bosch; The Netherlands; 6 Dept. of Gastroenterology, Meander Medical Center Amersfoort, PO 1502, 3800 BM, Amersfoort; The Netherlands; 7 Dept. of Surgery, Maastricht University Medical Center, PO 5800, 6202 AZ Maastricht; The Netherlands; 8 Dept. of Gastroenterology, VU Medical Center, PO 7057, 1007 MB Amsterdam; The Netherlands; 9 Dept. of Surgery, St.Elisabeth Hospital, PO 90151, 5000 LC Tilburg; The Netherlands; 10 Dept. of Surgery, Medical Center Alkmaar, PO 501, 1800 AM Alkmaar; The Netherlands; 11 Dept. of Gastroenterology, Onze Lieve Vrouwe Gasthuis, PO 95500, 1090 HM Amsterdam; The Netherlands; 12 Dept. of Surgery, Reinier de Graaf Gasthuis, PO 5011, 2600 GA Delft; The Netherlands; 13 Dept. of Surgery, Medical Center Leeuwarden, PO 888, 8901 BR Leeuwarden; The Netherlands; 14 Dept. of Surgery, University Medical Center Groningen, PO 30001, 9700 RB Groningen; The Netherlands; 15 Dept. of Surgery, St Antonius Hospital Nieuwegein, PO 2500, 3430 EM Nieuwegein; The Netherlands; 16 Dept. of Surgery, Leiden University Medical Center, PO 9600, 2300 RC Leiden; The Netherlands; 17 Dept. of Surgery, Amphia Hospital, PO 90158, 4800 RK Breda; The Netherlands; The Netherlands; 18 Dept. of Gastroenterology, Rijnstate Hospital, PO 9555, 6800 TA Arnhem; The Netherlands; 19 Dept. of Gastroenterology, Canisius Wilhelmina Hospital, PO 9015, 6500 GS Nijmegen; The Netherlands; 20 Dept. of Gastroenterology, Isala Clinics, PO 10400, 8000 GK, Zwolle; The Netherlands; 21 Dept. of Gastroenterology, St Antonius Hospital Nieuwegein, PO 2500, 3430 EM Nieuwegein; The Netherlands; 22 Dept. of Gastroenterology, Hospital Gelderse Vallei Ede, PO 9025, 6710 HN Ede; The Netherlands; 23 Dept. of OR/Evidence Based Surgery, Radboud University Nijmegen Medical Centre, PO 9101, 6500 HB Nijmegen; The Netherlands

## Abstract

**Background:**

In predicted severe acute pancreatitis, infections have a negative effect on clinical outcome. A start of enteral nutrition (EN) within 24 hours of onset may reduce the number of infections as compared to the current practice of starting an oral diet and EN if necessary at 3-4 days after admission.

**Methods/Design:**

The PYTHON trial is a randomised controlled, parallel-group, superiority multicenter trial. Patients with predicted severe acute pancreatitis (Imrie-score ≥ 3 or APACHE-II score ≥ 8 or CRP > 150 mg/L) will be randomised to EN within 24 hours or an oral diet and EN if necessary, after 72 hours after hospital admission.

During a 3-year period, 208 patients will be enrolled from 20 hospitals of the Dutch Pancreatitis Study Group. The primary endpoint is a composite of mortality or infections (bacteraemia, infected pancreatic or peripancreatic necrosis, pneumonia) during hospital stay or within 6 months following randomisation. Secondary endpoints include other major morbidity (e.g. new onset organ failure, need for intervention), intolerance of enteral feeding and total costs from a societal perspective.

**Discussion:**

The PYTHON trial is designed to show that a very early (< 24 h) start of EN reduces the combined endpoint of mortality or infections as compared to the current practice of an oral diet and EN if necessary at around 72 hours after admission for predicted severe acute pancreatitis.

**Trial Registration:**

ISRCTN: ISRCTN18170985

## Background

In 20% of patients, acute pancreatitis is complicated with peripancreatic and pancreatic necrosis [1]. In about 30% of these patients, this necrotic tissue becomes infected and this is associated with a mortality rate of approximately 15% [2,3]. In addition to infected necrosis, other infections such as bacteraemia and pneumonia frequently occur with a major negative impact on outcome [4,5]. Attempts to reduce infections with the prophylactic use of antibiotics were unsuccessful [6,7]. The current hypothesis is that these infections originate from the gut. Gut-derived bacteria translocate due to a combination of pathophysiological events - disturbed gastrointestinal motility, bacterial overgrowth, reduction of arterial blood flow, increased permeability of the gastrointestinal mucosal barrier and bacterial translocation - leading to distant systemic infections, including infection of peripancreatic or pancreatic necrosis [8-11]. These phenomena already occur within a few hours after onset of symptoms and cause early infections [12-14]. This implies that there is only a very narrow time-window for reducing bacterial translocation and subsequent infections [5]. In clinical practice, patients with predicted severe acute pancreatitis are generally kept at a 'nil per mouth' regimen until the disease severity becomes clear. After a few days, some patients can start an oral diet while others require a nasojejunal catheter for enteral nutrition. This usually takes 3 to 4 days and the deleterious effects on the gastrointestinal tract already have taken place [1].

Enteral nutrition (EN) has the ability to maintain gut integrity, stimulate gut contractility and the release of immunomodulating agents and blood flow to the gut [15]. The maintenance of blood flow, preserved motility and gut mucosal integrity theoretically reduce bacterial overgrowth and bacterial translocation and thereby potentially reduces the number of infections. Furthermore, maintaining mucosal integrity reduces the release of inflammatory mediators, decreases oxidative stress and abates the systemic inflammatory response syndrome (SIRS) [16]. EN has already proven superior to parenteral nutrition in reducing mortality and infections in predicted severe pancreatitis [17]. In intensive care patients with other diseases, a meta-analysis of randomised trials demonstrated a reduction of infections following a very early start of enteral nutrition [18]. A 2009 systematic review suggested that also in patients with acute pancreatitis a very early start of EN improves outcome [19].

The PYTHON trial was designed to investigate whether a start of EN within 24 hours after admission counteracts the early deleterious effect of SIRS on bowel mucosa and reduces infections and mortality in predicted severe acute pancreatitis, as compared to a start of EN after 72 hours.

## Methods

### Design

The PYTHON trial is a randomised controlled, parallel-group, superiority multicenter trial. Patients will be randomly allocated to receive EN within 24 hours through a nasojejunal catheter or start after 72 hours of admission with an oral diet if tolerated or EN through a nasojejunal catheter if necessary. Patients with a primary episode of predicted severe acute pancreatitis are eligible for randomization.

The aim of the study is to investigate whether the clinical outcome can be improved by a very early start of EN, as compared to the current practice of an oral diet and EN if necessary, i.e. at around 72 hours after admission.

### Primary endpoint

The primary endpoint is a composite of mortality or infections (i.e. bacteraemia, pneumonia and infected necrosis; see Table 1 for definitions) occurring during hospital admission or within 6 months following randomisation. Re-admission within 10 days after initial hospital discharge is considered part of the index admission.

**Table 1:** Primary endpoint: definition of infections

Infection	Definition
Infected pancreatic necrosis	Positive culture of pancreatic or peripancreatic necrotic tissue obtained by means of fine-needle aspiration or from the first drainage procedure or operation, or the presence of gas in the fluid collection on contrast-enhanced CT.

Bacteraemia	Positive blood culture. For non-pathogens (eg. coagulase negative staphylococci) at least 2 samples have to be positive.

Pneumonia	Coughing or dyspnoea, radiography with infiltrative abnormalities, raised inflammatory variables and positive sputum culture. On the intensive care unit a positive endotracheal culture is mandatory.

### Secondary endpoints

Attenuation of the acute inflammatory response is hypothesized to be part of the beneficial effect of very early feeding on outcome thus secondary endpoints to assess such an effect are:

• Daily APACHE-II scores (see Additional file 1; Table S3), SIRS (presence, onset, duration, see Additional file 1; Table S4), CRP, and white cell count during the first week following randomisation.

• The individual components of the primary endpoint, need for and number of interventions (percutaneous or endoscopic transgastric drainage, surgical or endoscopic necrosectomy, other operations), use of antibiotics, length of hospital stay and in-hospital mortality.

• New onset organ failure (single or multi-system organ failure, timing and duration of failure), daily Marshall scores and need for and duration of ICU admission at any time during follow-up (for definitions see Additional File 1; Table S1 and Table S2).

Other secondary outcome measures are:

• The presence of peripancreatic and pancreatic necrosis on contrast enhanced computed tomography (CT) and the CT Severity Index (CTSI) as measured 5-7 days after admission. Peripancreatic necrosis (only) is defined as persistent peripancreatic fluid collections on a CT performed more than 14 days after admission in the absence of pancreatic parenchymal necrosis.

• Abdominal pain as daily measured by the Visual Analogue Scale (VAS).

• Proportion of daily nutritional target achieved at 1 week after admission.

• Nutrition related complications: diarrhea, aspiration pneumonia, need for conversion from EN to PN or additional PN and need for replacement of nasojejunal feeding tube after dislocation.

• Number of days until intake of solid food.

• Number of patients without the need for tube feeding in the selective delayed EN Group.

• Proportion of cross-over between both study arms.

• Hand grip muscular strength measured by the Jamar dynamometer.

• Quality of life measured by the EQ5D and SF-36 questionnaires.

• Total direct medical and indirect costs from a societal perspective during admission and until 6 months after discharge (details are available in the Additional File 1).

### Study Population

**Figure 1:**
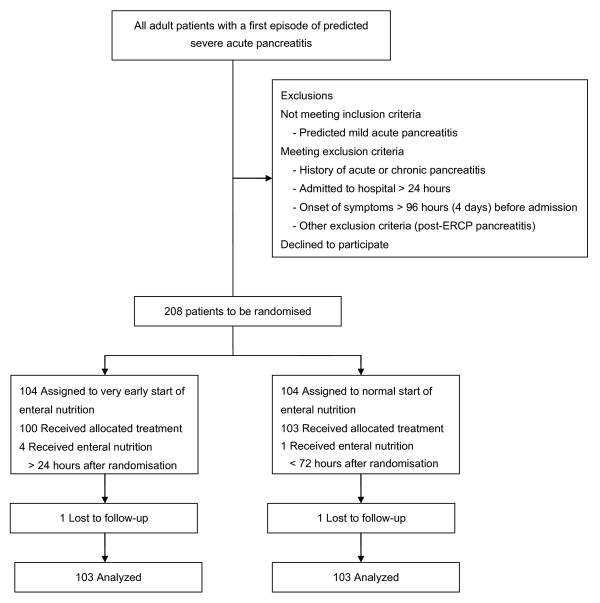
**Flow of participants PYTHON trial according to CONSORT**.

All patients admitted with a first episode of acute pancreatitis to one of the 20 participating hospitals of the Dutch Pancreatitis Study Group (DPSG, hospitals are listed below) will be assessed for eligibility within 24 hours after hospital admission. Potential eligible patients are followed during the first 24 hours after admission until eligibility is established or the first 24 hours following admission have passed. If patients are classified as 'predicted severe' acute pancreatitis, they are included and stratified for hospital and APACHE-II score <13 or ≥13 and randomised in a 1:1 ratio, to receive early EN or an oral diet or EN if necessary at 72 hours after admission (figure 1) [20].

### Inclusion criteria

• Diagnosis of acute pancreatitis if at least 2 of the 3 following features are present: 1) upper abdominal pain, 2) serum lipase or amylase levels above 3 times the upper level of normal and 3) characteristic findings of acute pancreatitis on cross-sectional abdominal imaging.

• Predicted severe pancreatitis within 24 hours after admission defined as one or more of the following (for definitions see Additional File 1; Tables S3 and S5):

- APACHE-II score ≥ 8

- Imrie-score ≥ 3

- CRP level > 150 mg/L

• Age ≥ 18 years

• Written informed consent

### Exclusion criteria

• History of acute or chronic pancreatitis

• Identification of patients > 24 hours after admission

• Onset of symptoms > 96 hours (4 days) before admission

• Acute pancreatitis due to malignancy or post-ERCP pancreatitis

• Diagnosis of acute pancreatitis confirmed during laparotomy for acute abdomen

• Artificial nutrition at admission (EN or PN)

• Pregnancy

### Treatment protocol

After the diagnosis has been established, the disease has been characterized as 'predicted severe' and written informed consent has been obtained, the patient will be randomised.

### Group A: Very early start of EN, i.e. within 24 hours

**Figure 2:**
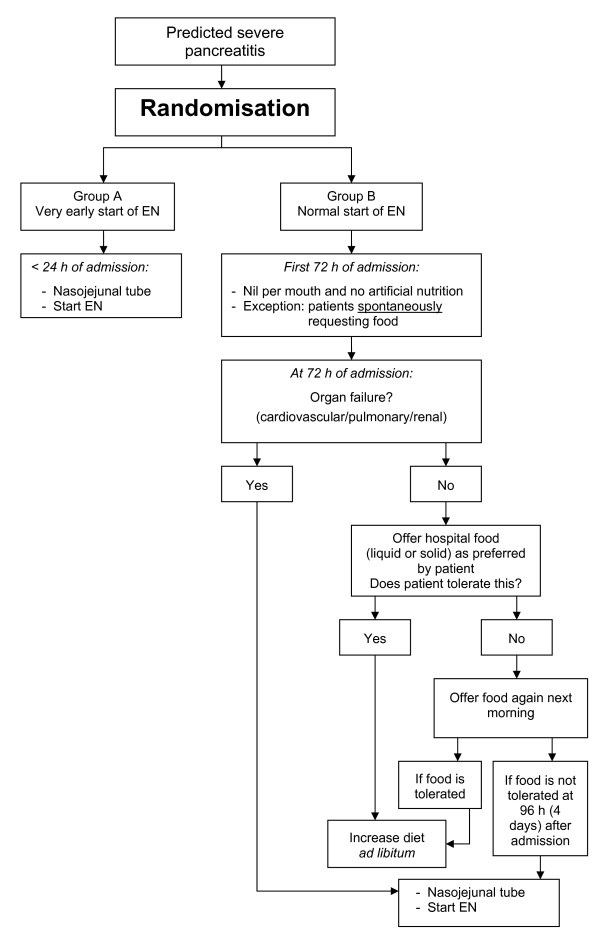
**PYTHON flowchart**.

A nasojejunal feeding tube is placed either endoscopically or radiologically. If placed endoscopically, an abdominal X-ray is performed to check the tube's position and in case of radiological placement, fluoroscopy is used. Nasojejunal placement is considered correct, when the tip of the tube is placed beyond Treitz' ligament. After tube placement (figure 2), EN is started immediately using a very strict volume regimen: 20 ml/h in the first 24 hours, 45 ml/h, between 24-48 h, 65 ml/h, between 48-72 hours and, at 72 hours and thereafter: full nutrition, defined as an energy target of 25 kcal/kg/day (ICU patients) and 30 kcal/kg/day (non ICU patients) [21,22].

### Group B: oral diet and EN if necessary at 72 hours after admission

Following randomisation, patients will be kept 'nil by mouth' without any artificial nutrition during the first 72 hours after admission (figure 2). If patients spontaneously request for oral food within these 72 hours, liquid and solid food are offered as requested and tolerated ("*ad libitum*"). If, at 72 hours after admission, patients develop organ failure, they will receive nasojejunal feeding with the same regimen as in group A. If, at 72 hours, there is no organ failure, patients are offered oral food *ad libitum*. If oral food is not tolerated, there is a re-challenge the next morning and if still not tolerated, EN is started through a nasojejunal feeding tube.

### General nutrition regimen

At 72 hours after the start of enteral feeding, the nutritional status will be evaluated and in case of intolerance, the type of EN will be changed accordingly (additional proteins, calories, fibers etc.). The patient is weighed twice a week during the first month after randomisation and once a week until discharge thereafter. If feeding is not tolerated EN is reduced to 50% and stepwise rebuilt gradually until tolerated. If, after two of such attempts, full nutrition can not be attained, PN will be started to reach the required energy target. Oral normal feeding is started, when abdominal pain has resolved and organ failure has subsided. In case of full tolerance of oral food nasojejunal feeding is gradually decreased. If pain relapses, EN is restarted. In case of nausea or vomiting, lowered consciousness (Glasgow Coma Score [GCS] 14 or lower in a non-intubated patient), or gastric residual volume (GRV) > 250 ml/6 hours, the position of the feeding tube is checked (see Additional file 1; figure S1).

### Patients in the Intensive Care Unit (ICU)

In case of ICU admission, irrespective of time from admission, the patient is fed according to the attending intensivist's preference (nasogastric or nasojejunal; enteral or parenteral). These patients are analyzed according to the treatment assigned.

### General treatment regimen

All patients will undergo contrast enhanced CT within 5-7 days after admission. Intravenous antibiotics are administered based on culture results and not as prophylaxis in case of necrotizing pancreatitis without documented infection. Invasive intervention for (suspected) infected necrosis is preferably postponed until the fluid collections are walled-off and demarcated on CT-scan. The type of antibiotics administered depends on local guidelines and is left to the discretion of the attending physician. ERCP is performed in case of suspected cholangitis or in case of biliary pancreatitis with clinically important persistent cholestasis. Patients can be discharged from hospital once abdominal pain has resolved, infection variables (leucocytes, CRP) have decreased to near normal and a normal oral diet is tolerated.

### Randomisation

Randomisation is performed centrally by the study coordinator using an internet randomisation module (Julius Center for primary care and health sciences, University Medical Center Utrecht, the Netherlands). Randomisation is stratified according to the predicted severity of disease ('predicted moderate severe' vs. 'predicted very severe'; APACHE-II < 13 or ≥ 13) and according to hospital. Permuted-block randomisation with varying block size is used and block size is concealed to all local investigators.

### Data collection and follow-up

Follow-up visits will take place at 3 and 6 months after discharge. Data collection will be performed by local physicians using a written case-record form. An independent auditor will check all primary and secondary endpoints and at least 10% of data in case-record forms against on-site source data. Discrepancies detected by the auditor will be resolved on the basis of consensus by two investigators unaware of the study-group assignment and not involved in patient care.

### Safety

An independent data and safety monitoring committee (DSMC) will evaluate the progress of the trial and will examine safety variables after every 25 patients have completed the 6 months follow-up. This evaluation is based on unblinded data, in the presence of the study coordinator as far as the 'explanatory part' of the meeting is concerned. After full explanation of the data presented, the study coordinator is dismissed and the DSMC discusses the consequences of the data presented. Adverse events are defined as 'any undesirable experience occurring to a subject during a clinical trial, whether or not considered related to the intervention' (i.e. early enteral nutrition). All involved physicians will repetitively be asked to report any potential adverse events. These adverse events will be listed and discussed with the DSMC. The outcome of the meeting of the DSMC will be discussed with the trial steering committee. The outcome will also be sent to the Utrecht institutional review board (IRB). The data of the deceased patients will be evaluated by the DSMC for cause of death and possible trial related serious adverse events. All possible adverse events will be reported to the Central Committee on Research involving Human Subjects (Centrale Commissie Mensgebonden Onderzoek [CCMO]) and the IRB using the online module https://toetsingonline.ccmo.nl 

### Ethics

The study will be performed in accordance with the declaration of Helsinki and the Dutch "Wet Medisch-wetenschappelijk Onderzoek met Mensen" (Medical Research Involving Human Subjects Act). The IRB of the University Medical Center Utrecht approved the protocol on the 4^th ^of March 2008. Secondary approval was sought from all local ethics committees. Informed consent will be obtained from each participating patient in oral and written form prior to randomisation. The PYTHON trial is registered in the ISRCTN register with identification number ISRCTN18170985. After approval of the protocol, 2 amendments were approved by the ethics board. These amendments followed new regulations in the Netherlands for the reporting of (serious) adverse events. The content of both amendments is incorporated in this protocol.

## Statistical Aspects

### Sample size calculation

The PYTHON trial is a superiority trial, hypothesizing a reduction in the primary endpoint in favour of early EN as compared to the current practice of an oral diet and EN, if necessary at 72 hours after admission. Data from patients in the PROPATRIA trial are used to calculate sample size [23]. All patients in the placebo-arm of the PROPATRIA trial received EN via a nasojejunal feeding tube. Patients from the PROPATRIA trial that fulfilled the PYTHON inclusion criteria (predicted severe acute pancreatitis < 24 hours after admission) were evaluated. In these patients EN was initiated after a median of 3 days. This equals the standard group in the PYTHON trial. In 39% of these patients an infection (bacteraemia, pneumonia, infected necrosis) occurred. The mortality in the placebo arm of PROPATRIA was 6%. All studies included in a meta-analysis performed by our group on EN versus PN that initiated EN within 24 hours after admission were selected [17]. In these patients (receiving EN within 24 hours), infections were diagnosed in 22% and mortality was 5%. Therefore, the expected reduction in mortality is only 1%. When mortality and infections are combined in the primary composite endpoint, an absolute reduction of 18% is expected (from 40 to 22%). With 80% power, two-sided 5% alpha and 1% loss to follow-up the sample size was set at 208 patients [http://www.stat.ubc.ca/~rollin/stats/ssize/b2.html].

### Descriptive statistics

For dichotomous data, frequencies will be presented. Continuous data will be presented as mean and standard deviation or median and interquartile range. Baseline characteristics (all prior to randomisation) are: age, sex, body mass index, etiology of pancreatitis, American Society of Anesthesiologists (ASA) classification, co-morbidity (i.e. cardiovascular disease, pulmonary disease, chronic renal insufficiency or diabetes), predicted severity (APACHE-II, Imrie score, CRP, BUN), presence of organ failure or multi-system organfailure, Marshall score, amount of intravenous fluids administered prior to randomisation, time from onset of symptoms to randomisation, time from onset of symptoms to admission and number of referrals prior to randomisation.

### Analyses

All analyses will be according to the intention-to-treat approach in which all randomised patients are included, regardless of adherence to study protocol. After the last patient in the trial has completed follow-up, an adjudication committee blinded for treatment allocation will evaluate each patient using the raw data for the possible occurrence of the primary endpoint. Disagreements will be resolved in a plenary consensus meeting. Occurrences of the primary and secondary endpoints are compared between the treatment groups. Results are presented as risk ratios with corresponding 95% confidence intervals. A two-tailed P < 0.05 is considered statistically significant.

### Additional analyses

To evaluate potential differences in systemic inflammatory response after randomisation, the area under the curve of APACHE-II and Marshall scores from randomization to day 7 will be calculated and compared between treatment groups. A predefined subgroup analysis will be done for the occurrence of the primary endpoint in patients with an APACHE-II < 13 or an APACHE-II ≥ 13. The precise relation between the primary endpoint and the time between start of symptoms and start of EN will be analyzed using a generalized linear model with logarithmic link function and Bernouilli distribution, with independent variables time (between start of symptoms and start of EN), time*time, APACHE-II score prior to randomization, hospital and co-morbidity (i.e. cardiovascular disease, pulmonary disease, chronic renal insufficiency or diabetes) [24].

### Premature termination of the study

An interim-analysis is performed on the primary endpoint when 50% of patients have been randomised and have completed the 6 months follow-up. The interim-analysis is performed by an independent statistician, blinded for the treatment allocation. The statistician will report to the independent DSMC. The DSMC will have unblinded access to all data and will discuss the results of the interim-analysis with the steering committee in a joint meeting. The steering committee decides on the continuation of the trial and will report to the central ethics committee. The Peto approach is used: the trial will be ended using symmetric stopping boundaries at P < 0.001 [25]. The trial will not be stopped in case of futility, unless the DSMC during the course of safety monitoring advices otherwise. In this case DSMC will discuss potential stopping for futility with the trial steering committee.

### Recruiting success

The trial was registered on the 28^th ^of February 2008 in the ISRCTN register. The first patient was randomised on the 17^th ^of August 2008. So far, 142 patients have been randomised and inclusion is ahead of schedule.

## Discussion

The PYTHON trial is designed to answer the question whether a very early start of EN will lead to a reduction of mortality or infections in patients with predicted severe acute pancreatitis. Over the years several strategies were found ineffective in improving the outcome for patients with severe pancreatitis [6,23,26]. The only strategy that was consistently found to be effective in preventing complications in patients with acute pancreatitis is EN [17]. This positive effect might be expanded with the use of very early EN. In a meta-analysis of randomised controlled trials in critically ill patients, a very early start of enteral nutrition reduced the rate of infections [18]. In acute pancreatitis, timing of EN has not been investigated in an adequately powered and well-designed randomised trial. Therefore, the answers provided by the PYTHON trial are most needed.

Finally, the costs of EN through a feeding tube versus an oral diet and only a feeding tube if necessary will be compared. The extra costs of employing a feeding tube in every patient might be compensated by an improvement in outcome and subsequent reduced costs.

### Rationale for design

A double-blind placebo-controlled design is generally the preferred design for a randomised trial. In this study, this type of design would not have been possible. In current practice in patients with acute pancreatitis a 'step-up' approach is used, i.e. an oral diet is offered and only if an oral diet is not tolerated, EN through a nasojejunal tube is initiated. In the early EN group of this study, all patients require a nasojejunal feeding tube to initiate effective early enteral nutrition in the small bowel. It is not possible to blind patients for an oral diet or a feeding tube. It would have been possible to place a feeding tube in all patients and to subsequently randomise between an immediate start in the early EN group versus an oral diet or EN after 72 hours in the control group. However, a radiological or endoscopical placement of a nasojejunal feeding tube is potentially aggravating for patients. In addition, having a feeding 'in situ' for 72 hours (without using it for EN), is inconvenient for patients.

To create substantial time difference between interventions, a time interval of 72 hours was chosen. Perhaps a wider time-interval, for instance 96 hours, would have created a larger difference between interventions. However, in the Netherlands, a prolonged starvation period for more than 72 hours is seldom applied and therefore not representative for current practice [27]. The primary endpoint is a composite endpoint of mortality or infections (bacteraemia, infected pancreatic or peripancreatic necrosis, pneumonia). This composite endpoint was chosen for two reasons. If the trial would be adequately powered to show a reduction in mortality alone, the sample size would constitute more than 8 thousand patients. This number of patients was not deemed feasible. In addition, previous studies have shown that infected necrosis, pneumonia and bacteremia have a high impact on prognosis of patients with acute pancreatitis [4,5].

The current design was chosen because of these methodological and ethical reasons.

## Conclusion

The PYTHON trial is a randomised controlled multicenter trial designed to show a reduction in the composite primary endpoint of mortality or infections following a very early start of EN compared with a normal start of EN in patients with predicted severe acute pancreatitis.

## Abbreviations

AP: Acute Pancreatitis; APACHE: Acute Physiology and Chronic Health Evaluation; ASA: American Society of Anesthesiologists; CCMO: Central Committee on Research Involving Human Subjects (in Dutch: Centrale Commissie Mensgebonden Onderzoek); CECT: Contrast enhanced Computed Tomography; CRP: C-Reactive Protein; CTSI: Computed Tomography Severity Index; DPSG: Dutch Pancreatitis Study Group; DSMC: Data Safety Monitoring Committee; EN: Enteral Nutrition; ERCP: Endoscopic Retrograde Cholangiopancreaticography; GCS: Glasgow Coma Scale; GRV: Gastric residual Volume; IC: Informed Consent; ICU: Intensive Care Unit; IRB: Institutional review board; PN: Parenteral Nutrition; SIRS: Systemic Inflammatory Response Syndrome; VAS: Visual Analogue Scale; WMO: Medical Research Involving Human Subjects Act (in Dutch: Wet Medisch-wetenschappelijk Onderzoek met Mensen)

## Competing interests

The authors declare that they have no competing interests.

## Authors' contributions

OJB drafted the manuscript. HCVS, SB, MGHB and HGG co-authored the writing of the manuscript. OJB, HCVS, MGHB, UAA, VBN, MAB, TLB, CHCD, HVG, BVR, AFMS, BJMW and HGG participated in the design of the study during several meetings of the Dutch Pancreatitis Study Group. OJB and HCVS performed the sample size calculation. All authors edited the manuscript and read and approved the final manuscript.

## Supplementary Material

Additional file 1 **supplementary appendix to the PYTHON study protocol**. Supplementary file containing information on the definition of organ failure, Marshall score, APACHE-II score, Systemic Inflammatory Response Syndrome Criteria, Imrie score, Cost analysis and a flowchart 'Suspicion of dislocated nasojejunal feeding tube'. Click here for file
