# Supplementary material for: Pancreatitis, very early compared with normal start of enteral feeding (PYTHON trial): design and rationale of a randomised controlled multicenter trial
Source: Trials. 2011 Mar 10;12:73. doi: 10.1186/1745-6215-12-73 (PMC3068962; doi:10.1186/1745-6215-12-73)
Supplement: Additional file 1 — supplementary appendix to the PYTHON study protocol. Supplementary file containing information on the definition of organ failure, Marshall score, APACHE-II score, Systemic Inflammatory Response Syndrome Criteria, Imrie score, Cost analysis and a flowchart 'Suspicion of dislocated nasojejunal feeding tube'. [file 1745-6215-12-73-S1.DOC]

**SUPPLEMENTARY INFORMATION TO THE PYTHON STUDY PROTOCOL**

# Table S1: Definitions of organ failure

The definitions of organ failure have been adapted from the most recent version of the revised Atlanta classification (http://www.pancreasclub.com/resources/AtlantaClassification.pdf).

Three organ systems will be assessed to define organ failure: respiratory, cardiovascular, and renal.

- Organ failure is defined using the Marshall scoring system (see below) as a score > 2 for at least one of these three organ systems: respiratory (pO2/FIO2); renal (serum creatinine in μmol/l or mg/dl); and cardiovascular (systolic blood pressure in mm Hg).
- Persistent single organ failure is defined as any organ failing over a 3-day period.
- Persistent multi-system organ failure is defined as two or more organs failing over the same 3-day period.

|  |  |  | **Score** |  |  |
| --- | --- | --- | --- | --- | --- |
| **Organ system** | 0 | 1 | 2 | 3 | 4 |
| Respiratory (PaO_2_/FIO2) | > 400 | 301-400 | 201-300 | 101-200 | < 100 |
| Renal*  (serum creatinine, µmol/l)  (serum creatinine, mg/dl) | < 134  < 1.4 | 135-169  1.4-1.8 | 170-310  1.9-3.6 | 311-439  3.7-4.9 | > 439  > 4.9 |
| Cardiovascular (systolic blood pressure, mmHg) | > 90 | < 90  Fluid responsive | < 90  Not fluid responsive | < 90, pH < 7.3 | < 90, pH < 7.2 |

Table S2: For non-ventilated patients, the FiO_2_ can be estimated from below:

| **Supplemental Oxygen (l/min)** | **FiO_2_** |
| --- | --- |
| Room air | 21% |
| 2 - 3 | 25% |
| 4 - 5 | 30% |
| 6 - 8 | 40% |
| 9 - 10 | 50% |

Considerations should be taken for patients with pre-existent chronic renal failure (baseline serum creatinine ≥ 134 μmol/l or ≥ 1.4 mg/dl).

For patients with the need for inotropic catecholamine support, a Marshall score of 2 is appointed for cardiovascular system and dependent on the pH a Marshall score of 3 or 4 can be appointed.

**Table S3: Acute Physiology and Chronic Health Evaluation II (APACHE II) scoring system [28]**

| **Physiological variable** | **High abnormal** | | | |  | **Low abnormal** | | | |
| --- | --- | --- | --- | --- | --- | --- | --- | --- | --- |
|  | **+4** | **+3** | **+2** | **+1** | **0** | **+1** | **+2** | **+3** | **+4** |
| Temperature (°C) | ≥ 41 | 39.0-40.9 |  | 38.5-38.9 | 36-38.4 | 24-35.9 | 32-33.9 | 30-31.9 | ≤ 29.9 |
| Mean Arterial Pressure  (mm Hg) | ≥ 160 | 130-159 | 110-129 |  | 70-109 |  | 50-69 |  | ≤ 49 |
| Heart rate | ≥ 180 | 140-179 | 110-139 |  | 70-109 |  | 55-69 | 40-54 | ≤ 39 |
| Respiratory rate | ≥ 50 | 35-49 |  | 25-34 | 12-24 | 10-11 | 6-9 |  | ≤ 5 |
| Oxygenation  FiO2 ≥ 0.5, record A-aDO_2_  FiO2 < 0.5, record PaO_2_ | ≥ 500  - | 350-49  - | 200-349  - | -  - | < 200  > 70 | -  61-70 | -  - | -  55-60 | -  ≤ 55 |
| Arterial pH | ≥ 7.7 | 7.6-7.69 |  | 7.5-7.59 | 7.33-7.49 |  | 7.25-7.32 | 7.15-7.24 | ≤ 7.14 |
| Serum sodium (mmol/l) | ≥ 180 | 160-179 | 155-159 | 150-154 | 130-149 |  | 120-129 | 111-119 | ≤ 110 |
| Serum potassium (mmol/l) | ≥ 7 | 6.0-6.9 |  | 5.5-5.9 | 3.5-5.4 | 3-3.4 | 2.5-2.9 |  | ≤ 2.4 |
| Serum creatinine (μmol/l)  Double score for AKI | ≥ 302 | 169-301 | 125-168 |  | 53-124 |  | ≤ 52 |  |  |
| Haematocrit (%) | ≥ 60 |  | 50-59.9 | 46-49.9 | 30-45.9 |  | 20-29.9 |  | ≤ 19 |
| White blood cell count (total/mm^3^) | ≥ 40 |  | 20-39.9 | 15-19.9 | 3-14.9 |  | 1-2.9 |  | ≤ 0.9 |
| 15 - Glasgow coma scale |  |  |  |  |  |  |  |  |  |
| Total Acute Physiology score (APS) | | | | | | | | | |
| Age points | < 44  45-54  55-64  65-74  ≥ 75 | 0  2  3  5  6 | Chronic Health points:  If the patient has a history of severe organ system insufficiency or is immunocompromised, assign points as follows:  For non-operative or emergency postoperative patients, 5 points  For elective postoperative patients, 2 point | | | | | | |
| APACHE-II score  (sum of APS + age points + chronic health points) | | | A-aDO_2_, alveolar-arterial oxygen tension difference; AKI, acute kidney injury; FiO2, fractional inspired oxygen; PaO_2_, arterial oxygen tension | | | | | | |

**Table S4: Individual Systemic Inflammatory Response Syndrome (SIRS) Criteria**

| SIRS is defined as 2 or more of the following: | |
| --- | --- |
| Temperature | > 38 of < 36 degrees |
| Heart rate | > 90 beats/minute |
| Respiration | > 20/min or PaCO2 < 32 mm Hg |
| WBC | > 12.000/mm^3^, < 4000/mm^3^ or 10% immature bands |
| SIRS is defined as the presence of 2 or more SIRS criteria. WBC, white blood cell count. | |

**Table S5: Imrie score or modified Glasgow score [29]**

| One point for each item: | |
| --- | --- |
| Age | > 55 years |
| PO2 arterial | < 60 mm Hg |
| Albumine | < 32 g/l |
| Total calcium | < 2 mmol/l |
| Leukocytes | > 15 x 10 9/l |
| LDH | > 600 U/l |
| Glucose (non diabetics) | > 10 mmol/l |
| Ureum after rehydration | >16 mmol/l |

**METHODS**

*Costs*

Cost-minimization analysis will be used to determine economic differences between EN within 24 hours through a nasojejunal catheter compared with an oral diet if tolerated and if necessary EN through a nasojejunal catheter at 72 hours of admission. Costs will be estimated from a societal perspective [30]. Direct medical costs and indirect costs related to absence from work will be estimated during admission and 3 months follow-up. Primary data will be used to assess the use of health care resources. In addition, at 3 months after discharge, patients will fill out the validated Health and Labor questionnaire and a diary to capture additional resource use [31]. Costs will be assessed according to the Dutch guidelines for (pharmaco-)economic research [32]. Guideline unit costs will be used for ICU stay, hospital stay, medication (i.e. antibiotics during admission and antidiabetic medication and pancreatic enzymes during follow-up), visits to primary and outpatient health care clinicians, home care and admission to rehabilitation centers or nursing homes [32,33]. Unit costs for operations, radiological procedures, endoscopic procedures and microbiology diagnostics will be calculated at one of the university hospitals and will include all personnel costs, costs of materials, costs of equipment, and overhead costs. Productivity losses due to absence from paid work were calculated according to the cost friction method [34]. Costs per patient will be calculated by multiplying volumes of resource with unit costs [30]. All costs will be set at the year 2010 price level using the price index rate of the Dutch health care sector.

**Figure S1:** **Flowchart – Suspicion of dislocated nasojejunal feeding tube**

Pause nutrition and

check position nasojenunal feeding tube by abdominal X-ray

Nausea or

Vomiting or

Lowered consciousness

Position feeding tube?

No dislocation, position

Nasojejunal

Dislocated, position

Nasogastric or Nasoduodenal

Continue feeding regimen and

place additional nasogastric tube to measure GRV

GRV > 250 ml/6 hours

Perform gastric decompression

3 times a day

until GRV < 250 ml

GRV < 250 ml/6 hours

Remove

nasogastric tube

Reposition

Nasojejunal

feeding tube

endoscopically or radiologically

**Reference List for Additional File 1 (continued from main manuscript)**

28. Knaus WA, Draper EA, Wagner DP, Zimmerman JE: **APACHE II: a Severity of Disease Classification System**. *Crit Care Med* **1985**, 13:818-829.

29. Corfield AP, Cooper MJ, Williamson RC, Mayer AD, McMahon MJ, Dickson AP, Shearer MG, Imrie CW: Prediction of Severity in Acute Pancreatitis: **Prospective Comparison of Three Prognostic Indices.** *Lancet* **1985**, 2:403-407.

30. Gold MR, Siegel JE, Russel LB. ***Cost-effectiveness in Health and Medicine***. New York, NY: Oxford University Press, 1996.

31. Van Roijen L, Essink-Bot ML, Koopmanschap MA, Bonsel G, Rutten FF. **Labor and health status in economic evaluation of health care. The Health and Labor Questionnaire**. *Int J Technol Assess Health Care* **1996**, 12:405-15.

32. Oostenbrink JB, Koopmanschap MA, Rutten FF. **Standardisation of costs: the Dutch Manual for Costing in economic evaluations**. *Pharmacoeconomics* **2002**, 20:443-54.

33. Oostenbrink JB, Buijs-Van der Woude T, van Agthoven M, Koopmanschap MA, Rutten FF. **Unit costs of inpatient hospital days**. *Pharmacoeconomics* **2003**, 21:263-71.

34. Koopmanschap MA, Rutten FF, van Ineveld BM, van RL. **The friction cost method for measuring indirect costs of disease**. *J Health Econ* **1995**, 14:171-89.
